# Comparison data of a two-target real-time PCR assay with and without an internal control in detecting *Salmonella enterica* from cattle lymph nodes

**DOI:** 10.1016/j.dib.2018.04.051

**Published:** 2018-04-22

**Authors:** Jianfa Bai, Valentina Trinetta, Xiaorong Shi, Lance W. Noll, Gabriela Magossi, Wanglong Zheng, Elizabeth P. Porter, Natalia Cernicchiaro, David G. Renter, Tiruvoor G. Nagaraja

**Affiliations:** a Kansas State Veterinary Diagnostic Laboratory, College of Veterinary Medicine, Kansas State University, Manhattan, KS 66506, Unites States; b Department of Diagnostic Medicine/Pathobiology, College of Veterinary Medicine, Kansas State University, Manhattan, KS 66506, United States; c Food Science Institute, College of Agriculture, Kansas State University, Manhattan, KS 66506, United States; d Yangzhou University College of Veterinary Medicine, Yangzhou, Jiangsu, China

**Keywords:** Real-time PCR, Threshold cycle, Internal control, Salmonella, Foodborne pathogen, Lymph node

## Abstract

A real-time PCR (qPCR) assay targeting on *inv*A and *pag*C genes was developed and validated for the detection and quantification of *Salmonella enterica* strains (Bai et al., 2018) [1]. A host gene, normally an endogenous housekeeping gene (Beer-Davidson et al., 2018; Poon et al., 2004) [2,3], or an irrelevant exogenous gene (Cheng et al., 2015; Sedlak et al., 2014) [4,5] has been widely used as an internal control to monitor nucleic acid extraction efficiencies and potential PCR inhibitions in PCR-based detection assays. An endogenous internal control designed based on the 18S rRNA gene was used in the above-mentioned qPCR assay. This 18S rRNA internal control amplifies the target gene in multiple species including bovine, swine, ovine, caprine and cervine. Data was generated by the duplex qPCR assay on 138 enriched cattle lymph node samples without the internal control, and compared with data on the same samples tested by the triplex qPCR assay that has the 18S rRNA gene as internal control. Threshold cycle (Ct) data for the duplex and the triplex qPCR on the 138 samples were similar, and are presented in this brief report.

**Specifications table**

**Table:** 

Subject area	*Biology*
More specific subject area	*Foodborne pathogen detection*
Type of data	*Table*
How data was acquired	*Real-time PCR data generated by a Bio-Rad CFX96 Touch™ Real-Time PCR Detection System*
Data format	*Threshold cycle (Ct) values generated and exported from the machine software*
Experimental factors	*Cattle lymph node samples were culture-enriched prior to detection*
Experimental features	*Samples were tested by a two-target real-time PCR and compared with and without an internal control*
Data source location	*Cattle were procured from Texas, Oklahoma, Kansas, and South Dakota*
Data accessibility	*All data is accessible from this article*

**Value of the data**

• An internal control was used and showed value in monitoring nucleic acid extraction efficiencies in a real-time PCR assay for *Salmonella enterica* detection.
• Similar threshold cycle (Ct) data were generated with and without the use of the 18S rRNA gene as internal control.
• Although an internal control is strongly recommended for real-time PCR assays in diagnostic settings, it may be optional to use for certain research projects like the one reported here.

## 1 Data

Real-time PCR (qPCR) test data on 138 culture-enriched cattle lymph node samples for *Salmonella enterica* detections is shown in Table 1. Data were generated by: 1) A duplex qPCR assay using *inv*A and *pag*C genes; and 2) A triplex qPCR assay using *inv*A, *pag*C, and 18S rRNA gene as an internal control. The Ct differences between the duplex and triplex qPCR assays are also presented in the last two columns in Table 1.

**Table 1:** Real-time PCR threshold cycle (Ct) data on 138 *Salmonella*-positive cattle lymph node samples with and without the 18S rRNA internal control.

**Sample ID**	**Three-gene qPCR data**			**Two-gene qPCR data**		**Ct difference (three-gene Ct minus two-gene Ct)**	
	*inv*A-FAM	*pag*C-VIC	18S-Cy5*	*inv*A-FAM	*pag*C-VIC	*inv*A	*pag*C
1	24.80	24.14	26.17	24.68	24.43	0.12	−0.29
2	25.50	24.91	27.22	25.02	24.74	0.48	0.17
3	25.58	24.82	27.39	25.42	25.09	0.15	−0.27
4	22.43	22.41	30.48	21.23	21.69	1.20	0.73
5	23.55	23.33	29.36	23.54	23.25	0.01	0.08
6	25.06	24.46	29.95	24.32	23.97	0.74	0.49
7	23.92	23.78	29.61	23.57	23.89	0.35	−0.11
8	27.64	26.88	28.50	29.73	28.47	−2.10	−1.59
9	26.37	25.70	24.41	26.91	26.05	−0.55	−0.35
10	27.27	26.34	25.36	26.58	25.98	0.69	0.36
11	26.74	26.01	27.84	26.18	25.65	0.56	0.36
12	27.50	26.68	27.26	29.01	28.31	−1.51	−1.64
13	25.87	25.33	26.50	28.59	27.62	−2.72	−2.29
14	28.59	27.79	28.56	26.71	26.49	1.89	1.30
15	25.28	24.62	25.62	26.13	25.70	−0.85	−1.08
16	28.28	27.16	27.85	27.07	26.74	1.21	0.42
17	27.98	27.18	27.80	27.04	26.69	0.94	0.49
18	28.14	27.56	27.46	27.54	27.66	0.60	−0.10
19	30.04	29.27	28.60	27.86	27.71	2.18	1.56
20	23.98	23.26	24.29	26.22	25.83	−2.24	−2.56
21	27.21	26.87	23.59	27.95	27.62	−0.75	−0.75
22	24.09	23.99	23.91	24.16	24.39	−0.07	−0.40
23	26.73	25.98	26.20	25.66	25.28	1.07	0.70
24	25.75	25.33	28.34	24.29	24.33	1.46	1.00
25	25.10	24.65	27.24	25.05	25.02	0.05	−0.37
26	26.07	25.37	25.68	28.40	27.54	−2.33	−2.17
27	27.92	27.14	27.08	29.11	28.27	−1.19	−1.13
28	27.65	26.64	27.29	27.09	26.49	0.55	0.15
29	26.31	25.42	25.85	26.09	25.55	0.23	−0.13
30	27.92	27.14	25.91	27.56	27.32	0.36	−0.18
31	26.04	25.13	25.25	25.34	24.79	0.71	0.34
32	26.59	25.75	27.14	26.18	25.86	0.41	−0.11
33	27.46	26.61	28.19	26.67	26.38	0.80	0.23
34	24.57	24.01	24.84	25.43	25.10	−0.86	−1.09
35	27.58	26.71	27.88	26.38	26.16	1.20	0.55
36	27.49	26.72	27.65	26.96	27.05	0.53	−0.33
37	27.17	26.32	28.33	26.52	26.27	0.64	0.05
38	26.77	26.04	28.07	25.99	25.69	0.79	0.34
39	26.31	25.25	24.99	28.39	27.45	−2.08	−2.20
40	28.26	27.21	27.35	28.13	27.29	0.13	−0.08
41	27.28	26.45	27.02	26.51	26.19	0.77	0.26
42	26.09	25.10	27.09	26.00	25.27	0.08	−0.17
43	27.29	26.26	28.34	26.10	25.49	1.20	0.77
44	27.32	26.21	28.40	26.39	25.80	0.92	0.41
45	28.07	27.17	30.25	26.87	26.37	1.20	0.79
46	26.56	25.39	28.37	26.02	25.08	0.54	0.31
47	23.15	22.23	23.49	24.35	23.80	−1.20	−1.57
48	26.55	25.57	27.67	26.92	26.33	−0.37	−0.76
49	25.98	25.12	27.51	25.17	24.59	0.81	0.53
50	27.60	26.50	29.31	27.34	26.48	0.25	0.03
51	28.33	27.25	27.04	27.40	26.69	0.93	0.56
52	27.23	26.21	28.26	26.32	25.69	0.91	0.52
53	27.08	25.82	28.87	26.46	25.65	0.62	0.16
54	28.26	27.15	29.04	27.12	26.31	1.14	0.84
55	25.68	24.70	27.15	27.84	26.91	−2.16	−2.21
56	21.52	21.04	28.62	21.74	21.87	−0.22	−0.83
57	26.34	25.29	28.65	26.58	25.83	−0.24	−0.54
58	27.61	26.38	29.39	27.39	26.66	0.22	−0.27
59	24.36	24.11	29.42	23.64	23.58	0.73	0.53
60	25.18	24.34	28.44	25.37	25.08	−0.19	−0.74
61	27.22	26.19	28.69	26.51	25.91	0.71	0.28
62	25.70	24.51	27.56	26.45	25.85	−0.76	−1.34
63	35.24	33.14	26.01	35.36	34.26	−0.12	−1.12
64	26.08	25.12	26.23	26.03	25.39	0.06	−0.27
65	27.54	26.41	26.71	27.19	26.36	0.34	0.05
66	27.01	26.01	28.38	27.22	26.73	−0.21	−0.72
67	26.25	25.17	28.33	26.51	25.87	−0.26	−0.70
68	25.47	24.77	27.59	25.36	25.50	0.11	−0.73
69	25.57	24.46	27.84	27.78	26.55	−2.20	−2.08
70	27.23	26.31	24.81	27.80	27.15	−0.56	−0.84
71	26.40	25.65	25.30	26.98	26.61	−0.58	−0.96
72	26.79	26.08	28.92	26.93	26.70	−0.14	−0.62
73	24.75	24.02	26.08	28.45	27.35	−3.70	−3.33
74	24.79	24.05	25.82	24.81	24.47	−0.02	−0.43
75	25.89	25.09	23.52	27.25	26.61	−1.36	−1.51
76	26.25	25.10	23.78	29.95	28.36	−3.70	−3.26
77	27.10	25.98	26.00	29.22	27.90	−2.12	−1.92
78	26.56	25.85	27.84	27.99	27.48	−1.43	−1.63
79	28.75	28.06	27.66	29.00	28.49	−0.25	−0.43
80	26.34	25.63	26.99	30.09	28.99	−3.75	−3.37
81	29.55	28.84	29.09	30.05	29.69	−0.50	−0.85
82	25.14	24.07	26.84	26.02	25.53	−0.88	−1.47
83	26.52	25.41	28.42	27.76	26.77	−1.24	−1.36
84	26.01	24.77	31.15	27.07	26.25	−1.06	−1.49
85	24.59	23.87	28.19	25.91	26.00	−1.32	−2.13
86	24.39	23.58	29.28	27.50	26.61	−3.12	−3.02
87	29.00	27.90	28.80	27.12	26.41	1.88	1.49
88	28.37	28.03	29.57	25.04	24.53	3.33	3.50
89	25.39	24.82	28.41	26.26	25.94	−0.87	−1.12
90	23.41	22.73	24.13	26.76	26.45	−3.35	−3.72
91	24.27	23.48	25.85	24.64	24.59	−0.20	−0.52
92	26.42	25.04	29.56	26.12	25.43	0.31	−0.39
93	25.94	24.51	27.12	28.04	26.45	−2.11	−1.94
94	23.30	22.74	27.24	22.86	22.82	0.44	−0.08
95	23.63	22.80	28.67	23.90	23.85	−0.27	−1.05
96	23.92	22.98	29.31	24.94	24.66	−1.02	−1.67
97	25.20	24.14	29.74	25.41	24.60	−0.21	−0.46
98	25.47	25.14	24.71	26.57	25.50	−1.10	−0.36
99	26.87	25.27	27.17	26.38	26.53	0.49	−1.26
100	30.85	29.21	26.74	27.58	25.64	3.27	3.57
101	21.05	21.13	23.39	21.47	22.27	−0.42	−1.14
102	24.37	23.67	25.67	27.25	26.81	−2.88	−3.14
103	19.95	20.06	26.32	20.04	20.22	−0.10	−0.16
104	25.18	24.41	25.14	28.58	27.52	−3.41	−3.12
105	25.11	24.52	27.82	25.70	25.65	−0.59	−1.14
106	26.62	25.76	27.45	25.99	26.03	0.63	−0.26
107	24.20	23.51	26.20	27.67	27.18	−3.47	−3.67
108	25.01	23.96	28.20	25.06	24.67	−0.05	−0.71
109	25.10	24.17	27.03	25.88	25.55	−0.78	−1.38
110	29.27	28.34	22.96	26.00	25.44	3.27	2.90
111	24.26	23.77	29.16	21.11	21.53	3.15	2.25
112	20.70	20.58	26.11	24.73	24.30	−4.03	−3.72
113	19.82	20.08	26.07	21.25	21.64	−1.43	−1.56
114	19.34	19.51	28.64	21.39	22.10	−2.05	−2.59
115	22.27	22.17	29.01	19.97	20.46	2.30	1.71
116	25.81	24.96	28.46	29.54	29.16	−3.72	−4.20
117	25.42	25.02	24.95	26.44	26.05	−1.03	−1.03
118	26.11	25.49	31.78	25.74	26.40	0.37	−0.91
119	26.54	25.33	29.62	27.03	26.74	−0.49	−1.41
120	26.10	25.37	23.27	27.33	26.64	−1.23	−1.27
121	28.90	28.02	24.40	27.25	26.87	1.65	1.15
122	31.25	30.99	21.84	31.58	30.26	−0.33	0.73
123	24.49	23.91	25.85	21.49	22.29	3.00	1.61
124	27.29	26.72	22.95	25.34	25.22	1.95	1.50
125	26.56	25.81	26.91	23.23	23.92	3.33	1.88
126	25.88	25.04	25.78	27.33	27.23	−1.45	−2.20
127	23.11	22.83	29.40	26.55	26.20	−3.44	−3.37
128	24.00	23.15	27.29	25.07	25.18	−1.07	−2.03
129	22.00	21.50	28.38	24.92	24.85	−2.92	−3.35
130	25.58	24.85	26.46	29.41	29.19	−3.82	−4.34
131	24.47	24.21	24.36	27.63	26.84	−3.16	−2.63
132	24.28	23.76	27.51	25.43	25.41	−1.15	−1.65
133	23.74	23.42	23.99	27.14	26.18	−3.41	−2.76
134	24.45	24.04	24.32	25.00	25.19	−0.55	−1.15
135	24.26	23.46	24.73	25.22	25.40	−0.96	−1.94
136	24.57	23.71	28.15	24.74	24.62	−0.17	−0.92
137	25.17	24.27	26.50	25.15	24.89	0.03	−0.62
138	25.73	24.92	27.30	25.95	25.60	−0.21	−0.68

^*^Relatively high Ct values for the 18S rRNA internal control gene was caused by dilutions of host cells during culture-enrichment.

## 2 Experimental design, materials and methods

A total of 647 subiliac lymph nodes were collected from cattle procured from Texas, Oklahoma, Kansas, and South Dakota [6] and transported in cold storage to the Kansas State University for processing within 24 h. Each lymph node was manually trimmed to remove fat and fascia tissues. Trimmed lymph nodes were surface sterilized by a 5 sec submersion in boiling water, placed in a sterile bag, then manually pulverized with a rubber mallet. Eighty milliliters of TSB was added to each bag and sterilized lymph nodes were then homogenized for 30 s in a Stomacher 80 Biomaster (Thomas Scientific, Swedesboro, NJ) prior to culture-enrichment. The enrichment procedure has been described [6], [7]. Briefly, the homogenate was incubated at 25 °C for 2 h then at 42 °C for 12 h. One milliliter of enriched homogenate was then subjected to immunomagnetic separation using 20 µl anti-*Salmonella* beads. One hundred microliters of PBS was added to the final immunomagnetic separation step. The bead suspension was then transferred into 3 mL RV broth and incubated at 42 °C for 18–20 h. One hundred microliters of enriched homogenate was streaked onto HE agar plates and incubated at 37 °C for 24 h. Six dark-colored colonies with morphology consistent with *Salmonella* were re-streaked onto BAPs and incubated at 37 °C for 18–20 h. The resulting cultures was used for DNA extraction by boiling 1 ml of culture for 10 min and centrifuging at 9300 g for 5 min; the supernatant was used as template for the qPCR reactions with the duplex qPCR assay using both *inv*A and *pag*C genes as molecular targets. Endogenous housekeeping genes [2], [3], or irrelevant exogenous gene [4], [5] have been widely used as internal controls to monitor nucleic acid extraction efficiencies and potential PCR inhibitions in PCR-based detection assays. We have selected an endogenous housekeepig gene, 18S rRNA gene, as internal control in this study. Randomly selected 138 duplex qPCR-positive samples were proceed with the triplex qPCR assay using the same molecular targets, and with an 18S rRNA gene as internal control [1]. Comparison data using the duplex and triplex assays is presented in Table 1.
