# Supplementary material for: Comparison data of a two-target real-time PCR assay with and without an internal control in detecting Salmonella enterica from cattle lymph nodes
Source: Data Brief. 2018 Apr 22;18:1819–24. doi: 10.1016/j.dib.2018.04.051 (PMC5998743; doi:10.1016/j.dib.2018.04.051)
Supplement: Supplementary file 1 — Supplementary material. [file mmc1.docx]

Conflict of Interest

All the authors declares on No conflict of Interest.
