# Supplementary material for: Comparison data of a two-target real-time PCR assay with and without an internal control in detecting Salmonella enterica from cattle lymph nodes
Source: Data Brief. 2018 Apr 22;18:1819–24. doi: 10.1016/j.dib.2018.04.051 (PMC5998743; doi:10.1016/j.dib.2018.04.051)
Supplement: Supplementary file 2 — Supplementary material. [file mmc2.docx]

College of Veterinary Medicine

Kansas State Veterinary Diagnostic Laboratory

**REVISED**

Manuscript No.: DIB-D-18-00823

Journal Title: Data in Brief

Title: Comparison data of a two-target real-time PCR assay with and without an internal control in detecting *Salmonella enterica* from cattle lymph nodes

Authors: Jianfa Bai, Valentina Trinetta, Xiaorong Shi, Lance W. Noll, Gabriela Magossi, Wanglong Zheng, Elizabeth P. Porter, Natalia Cernicchiaro, David G. Renter, and T. G. Nagaraja

Corresponding author: Jianfa Bai

April 9, 2018

Dear Editor,

All authors listed above declare that there is no conflict of interest to this piece of work described in this manuscript.

Sincerely yours,

Jianfa Bai, Ph.D., Associate Professor

Director of Molecular Research and Development

Kansas State Veterinary Diagnostic Laboratory

Dept. Diagnostic Medicine/Pathobiology

Kansas State University

2005 Research Park Cir

Manhattan, KS 66502

Phone: (785)-532-4332

Fax: (785)-532-3909

[jbai@vet.ksu.edu](mailto:jbai@vet.ksu.edu)
